# Zein Monolayers: Characterization and Interaction with (Bio)surfactants

**DOI:** 10.1021/acs.langmuir.5c02426

**Published:** 2025-07-23

**Authors:** Kamil Wojciechowski, Mario Campana, Agnieszka Samel, Emilia Baran

**Affiliations:** 1 Faculty of Chemistry, Warsaw University of Technology, Noakowskiego 3, Warsaw 00-664, Poland; 2 Department of Chemistry, University of Warmia and Mazury in Olsztyn, Pl. Łódzki 4, Olsztyn 10-721, Poland; 3 ISIS Pulsed Neutron and Muon Facility, Rutherford Appleton Laboratory, Didcot, Oxfordshire OX11 0QX, U.K.

## Abstract

Zein is the main protein of corn seeds, which is often employed in food packaging and as a model of keratin. In this study, zein monolayers were deposited from nonconventional solvents: aqueous ethanol and acetic acid, on pure water that was later exchanged for 1% (bio)­surfactant solutions: SDS, CTAB, Triton X-100, and the saponin-rich plant extracts of soapwort ( L.) and cowherb ( [P. Mill.] Rauschert), as well as Quillaja bark saponins (QBS). The monolayers on pure water could be reversibly compressed up to ∼47 mN/m. On the basis of neutron reflectivity (NR) results, the liquid expanded–liquid expanded (LE-LE) transition observed at π ≈ 30 mN/m was assigned to an expulsion of the well-packed monolayer initially located on the air side of the interface, toward the aqueous side. The phase transition was accompanied by an increase in the layer thickness (from ∼1 to ∼6 nm) and the adsorbed amount (from ∼1.7 to ∼5.0 mg/m^2^). In contrast to the saponin-rich solutions, the synthetic surfactants introduced to the subphase easily removed the zein monolayer precompressed to π_0_ = 30 mN/m, although the mechanism was different for the ionic (continuous displacement) and for the nonionic (orogenic-like). The zein layers at Si/water and their resistance to the detergent activity of SDS and QBS were assessed using NR, proving that the layers cast from acetic acid showed slightly higher mechanical strength than those cast from aqueous ethanol.

## Graphical abstract

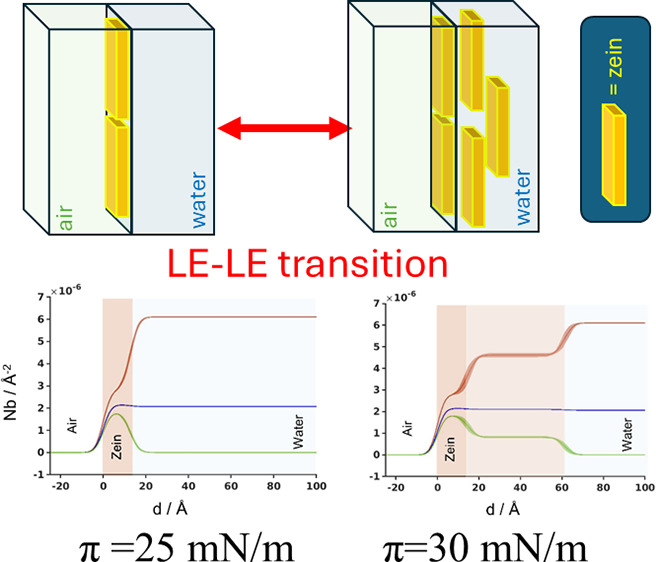

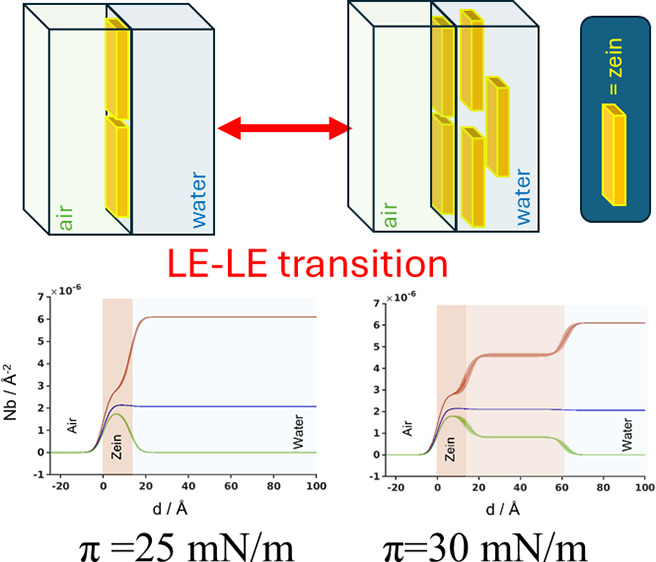

## Introduction

Zein is a group of prolamin (alcohol-soluble) storage proteins of corn (). Its main fraction consists of two groups of peptides: Z19 and Z22 known as α-zein (70–85%) and the other three being present at lower and variable concentrations: β-zein (1–5%), γ-zein (10–20%), and δ-zein (1–5%). The zein’s polypeptide chain is composed mainly of hydrophobic amino acid residues, like glutamine, leucine, alanine, or proline, which make up ∼60% of the molecule^1,2^ (see the Supporting Information for the zein’s amino acid sequence). The Z19 and Z22 peptides consist of 210 and 240 residues, respectively (Mw = 23–24 and 26–27 kDa).^3^ Zein is not soluble in water but can be dispersed in aqueous ethanol mixtures, highly alkaline solutions of pH >11, or acetic acid solutions. In aqueous-alcoholic and acetic acid solutions, the dominant secondary structure of zein is α-helical, but the protein self-assembly is accompanied by conversion into β-sheets, which then pack into stripes and further into rings, discs, and, eventually, spheres.^1,4^ The fraction of β-sheet structures is generally higher in aqueous-alcoholic solutions than in aqueous acetic acid ones and increases with the increasing percentage of the nonaqueous component.^1^ Depending on the employed experimental technique and the solvent composition, different researchers proposed different structural models, the most popular known as the Argos’, Garratt’s, and Matsushima’s models.^3,5,6^ The latter assumes formation of 9–10 homologous helical segments aligned antiparallelly, forming a ribbon. The hydrophobic groups are exposed on the sides of the ribbon, while the hydrophilic glutamine-rich bridges constitute the ribbon edges. In Argos’ and Garratt’s models, the antiparallel aligned helices cluster into a distorted cylinder, resulting in globular, rather than elongated structures. In all models, however, the zein molecule owes its amphiphilicity to spatial separation of the hydrophilic and hydrophobic segments, explaining its ability to self-assemble in solution.

Besides being a very promising natural component of food packaging films, the interest in zein arises also from its similarity to keratin, the major structural protein of skin, hair, and nails. Analogously to keratin, the tight packing of the native structure can be loosened by interaction with anionic surfactants (e.g., sodium dodecyl sulfate, SDS), resulting in hydrophilization and a consequent increased aqueous solubility of the surfactant-unfolded zein. This feature is a basis of the so-called “zein solubilization test”, where the amount of zein solubilized in a surfactant solution correlates well with the irritant potential of the surfactants.^7,8^

The first report on the zein monolayer dates back to 1937, where the maximum achieved surface pressure amounted to π = 17.7 mN/m.^9^ The low mean area per amino acid unit (12.5 Å^2^) and brightening of the monolayer observed under a dark-field microscope prompted Mitchell to assign this surface pressure value to the zein monolayer collapse. More recent studies achieved much higher surface pressure values exceeding 40 mN/m, with a quasi-plateau region around 25 mN/m, instead of a collapse. Pezron et al. and later Sousa et al. interpreted the quasi-plateau as an indication of either a monolayer–bilayer transition or a change in orientation of the helicoidal or unfolded chain portions.^2,10^ Zhang et al. transferred a zein monolayer cast from 80% aqueous solution of ethanol precompressed to π = 18 mN/m onto a glass plate and visualized it using AFM. On this basis, they concluded that the zein monolayer was composed of zein nanoparticles of 30–80 nm.^11^

In this paper, we studied in more detail the zein monolayers spread from aqueous ethanol and acetic acid solutions by analyzing their compression–decompression isotherms and neutron reflectivity (NR) in a Langmuir trough. A special focus was put on an unusual liquid expanded–liquid expanded (LE-LE) phase transition observed around a surface pressure of 30 mN/m, associated with a sudden increase in the monolayer thickness on the aqueous side. The layers spread on silicon wafers by spin-coating were characterized at the solid–liquid interface using NR and compared with the monolayers spread on the air–water interface. In the second step, the resistance of the zein layers spread on the water–air (Langmuir monolayers) and silicon–water (spin-coated multilayers) interfaces to several (bio)­surfactants (SDS, CTAB, Triton X-100, and saponin-rich extracts from Quillaja (QBS), cowherb, and soapwort) was assessed.

## Experimental Section

### Chemicals

Milli-Q water (Merck-Millipore, Molsheim, France) was used for all solutions if not stated otherwise. For some neutron experiments, either pure D_2_O (99.9% D, purchased from Sigma-Aldrich) or its mixtures with Milli-Q water were employed. Zein and synthetic surfactants sodium dodecyl sulfate (SDS), cetyltrimethylammonium bromide (CTAB), Triton X-100, and Quillaja saponins (QBS, 84510) were purchased from Sigma (Poznan, Poland). The saponin-rich plant extracts from the seeds of cowherb ( [P. Mill.] Rauschert) and the roots of soapwort ( L.) were prepared as described in ref 12.

### Langmuir Trough Experiments

For deposition of zein monolayers on the water–air interface, zein was dissolved in an ethanol–water (9:1, v/v) mixture and in glacial acetic acid, both at 1 mg/mL. The surface pressure compression isotherms (π­(*A*)) and relaxation curves after a quick compression, π­(*t*)*,* for the monolayer on pure water and on the (bio)­surfactant solutions were recorded using a home-built Langmuir trough equipped with a Wilhelmy plate made of filter paper (ashless Whatman Chr1) connected to an electrobalance (KSV, Finland). The subphase temperature was controlled by means of a thermostat. The experimental details are given in ref 13. Briefly, the trough with a total area of 194.25 cm^2^ equipped with two connection ports for the subphase exchange with minimum distortion of the monolayer was used. The appropriate zein solution was deposited onto a Milli-Q water subphase with a Hamilton micro syringe and left for evaporation during 15 min. The monolayer was compressed at a rate of 7 mm/min to reach the given value of surface pressure in order to record the compression isotherm (π­(*A*)) and compressibility modulus ( $C_{s}^{-1}$ ­(π)) (eq 1).

$$C_{s}^{-1}=-\frac{d\pi }{dA}\cdot A$$ 1

where *A* is the mean molecular area at a given surface pressure, π. In monolayer relaxation experiments, after the monolayer deposition and solvent evaporation, the monolayer was compressed to π_0_ = 30 mN/m at a rate of 7 mm/min, and the subphase exchange procedure was initiated. For this purpose, a peristaltic pump with a flow rate of 9 mL/min was used. Immediately before starting the Langmuir trough experiments with the subphase exchange, the (bio)­surfactants were dissolved in Milli-Q water to achieve the concentration of 2.5% (w/w) of the dry mass or of the active ingredient, which after dilution with the water from the trough during the subphase exchange resulted in the final concentration of 1%. The subphase exchange was complete after 900 s, but surface pressure was monitored for 6000 s. The experiments with the respective Gibbs layers (without the zein monolayer) were performed analogously, omitting the monolayer deposition and compression steps. At the end of each monolayer relaxation measurement, the surface compression (dilational) modulus, *E*, of the monolayer, was probed by performing oscillatory movements of the barriers. A frequency of 0.1 Hz and a relative amplitude of 2% were used.

### Spin-Coated Zein Layers on Si

For deposition of zein on Si blocks, two 0.5% (w/w) zein solutions were prepared, one in glacial acetic acid (zein_A) and one in an ethanol–water (9:1, v/v) mixture (zein_E). They were left overnight and filtered through a 0.45 μm syringe filter. While 10 mL of zein_A could be filtered easily, the aqueous ethanol solution clogged the filter already after passing ∼2 mL, pointing to the presence of a significant amount of aggregates >0.45 μm. Single-crystal (111) silicon substrates were cleaned with a “piranha solution” consisting of a 9:1 mixture of H_2_SO_4_/H_2_O_2_. The blocks were heated and maintained between 80 and 90 °C for 10 min, then rinsed with Milli-Q water, and stored in water prior to use. One mL of each filtered zein solution was deposited on Si blocks and spin-coated following the protocol described by Shi et al.:^14^ 4000 rpm for 80 s for zein_E and 800 rpm for 20 s followed by 3000 rpm for 20 s for zein_A using a Laurell WS-650-23B spin-coater. The blocks were then dried overnight in a vacuum oven at 50 °C to remove residual solvent.

### Neutron Reflectivity

Neutron reflectometry was used to study the structure of zein layers at both the air/liquid and solid/liquid interfaces. Measurements at the air/liquid interface were performed using an INTER reflectometer^15^ at ISIS, RAL, Didcot, UK. The wavelength range on the INTER reflectometer was between 1.5 and 16 Å; measurements were performed at 0.8 and 2.3°. Reflectivity, defined as the ratio between the specularly reflected neutrons and those in the incident beam, was calibrated using D_2_O. Measurements were performed using D_2_O, zein-matched water (ZMW, 39.4% D_2_O–60.6% H_2_O), and null-reflecting water (NRW, 8.2% D_2_O–91.8% H_2_O). An additional measurement at 0.5° was performed for ZMW in order to visualize the critical edge at low *Q*. The three contrasts used were cofitted to a single model, but it is worth mentioning which information each can deliver. When measuring at the air/NRW interface, the reflectivity selectively arises from the layer adsorbed at the interface and is proportional to the adsorbed amount at the interface Γ (eq 2):

$$\Gamma =d\rho \frac{{\mathrm{Nb}}_{\mathrm{layer}}}{{\mathrm{Nb}}_{\mathrm{zein}}}$$ 2

where ρ is the density of zein (a value of 1.40 g/mL was used in H_2_O and adjusted for H/D exchange),^16^ *d* and Nb_layer_ are the experimentally determined layer thickness and scattering length density (SLD) of the layer, and Nb_zein_ is the SLD of zein calculated from its average composition^17^ (Nb_zein_ = 1.74 × 10^–6^ Å^–2^ in H_2_O and 2.79 × 10^–6^ Å^–2^ in D_2_O, changing linearly with bulk composition). Measurements with ZMW are sensitive to the penetration of the protein layer within the aqueous phase: as neutrons cannot distinguish between the protein and the solvation water, changes in reflectivity compared to the bare interface must be attributed to the layer exposed to air.^18^ Lastly, the D_2_O contrast is the most sensitive to the overall interfacial structure.

All measurements were performed by using a Langmuir trough enclosed in a box fitted with quartz windows to limit the extent of evaporation. To enable the unimpeded passage of the neutron beam, the barrier position was calibrated to allow an area between 168 and 710 cm^2^. This gave a compression ratio of ∼4, not enough to cover the whole isotherm but sufficient to pass from π ∼18 mN/m to π >30 mN/m. In all cases, enough material to reach a surface pressure between 18 and 20 mN/m was spread on the surface. Compression was started approximately 15 min after spreading to allow solvent evaporation and monolayer equilibration. Measurements were taken at fixed surface pressures of π = 20, 25, and 30 mN/m. An additional measurement was taken upon expansion at π = 25 mN/m to check for possible hysteresis.

The measurements at the solid/liquid interface were performed using an OFFSPEC reflectometer^19^ at ISIS, RAL, Didcot, UK.^20^ OFFSPEC is a time-of-flight reflectometer with a wavelength range between 2 and 14 Å; to cover a sufficiently broad *Q* range, measurements were performed using two incident angles, 0.6 and 2.3°. Reflectivity was calibrated using D_2_O. Measurements were then performed both in D_2_O and H_2_O, and the resulting reflectivity profiles were cofitted to a single model. The silicon oxide was measured against D_2_O prior to the spin-coating and was cofitted along with the two contrasts to constrain the characterization of the oxide layer. Background was subtracted using the pixels in the area detector away from the incident beam. The flow cell used in the experiment was supplied by ISIS and allowed the exchange of the aqueous subphase by means of an HPLC pump.

### Zein Solubilization Test

A zein solubilization test was performed according to a protocol described in our previous study^12^ based on a modified Götte protocol.^21^ Briefly, 0.5 g of zein was weighed into a 10 mL vial, which was then filled with 10 mL of 1% surfactant or plant extract solution. The content was stirred for 1 h at 35 °C and subsequently centrifuged at 5000 rpm before dry mass content determination using an Axis ATS60 moisture analyzer (105 °C). Analogously, the blank experiments were performed using Milli-Q water (to determine solubility of zein in water), and the dry mass was determined in 1% surfactant and extract solutions. All tests were performed in triplicate. The zein solubility in 1% surfactant or plant extract solutions was calculated after subtracting the dry mass of zein soluble in pure water and the dry mass of the surfactant/extract solution.

## Results and Discussion

### Zein Monolayer Characterization

In order to characterize the zein monolayers and to investigate the effect of a spreading solvent on their structure, two types of monolayers were spread on the surface of Milli-Q water using an ethanol–water mixture (9:1 v/v) and glacial acetic acid (Figure 1). Following the approach of Mitchell,^9^ the deposited protein amount is presented as a mean molecular area per amino acid unit, using the average molecular weights of 27 kDa and 113 Da, for the zein molecule and an amino acid, respectively. This results in 239 amino units per zein molecule.^2^ Even though the employed solvent mixtures were not typical spreading solvents, both compression isotherms provided a similar picture of a single phase transition around π = 25 mN/m, in line with the previously reported isotherms spread from, e.g., a chloroform–methanol mixture (9:1 v/v).^10^ Both monolayers could be compressed up to 47 mN/m without any collapse. This value is higher than the highest surface pressure reported in previous studies, where compression was stopped at π = 27–43 mN/m.^2,10^ The phase coexistence region was wider and flatter for the ethanol–water monolayer, pointing to some effect of the spreading solvent on the observed phase transition. The compression–decompression isotherms for both depositing solvents showed little hysteresis upon decompression down to π ≈ 25 mN/m, confirming that the zein monolayers did not collapse even upon compression to π = 47 mN/m. However, the decompression isotherm started to deviate from the compression branch for surface pressures below ∼25 mN/m. During the decompression of the monolayer cast from acetic acid, a kink in the isotherm could be observed upon approaching π = 30 mN/m, while for the ethanol–water, the transition was smoother. Since the monolayer collapse could be excluded (lack of hysteresis at higher compressions), this behavior may be related to some kinetic limitations of the phase transition process. The latter apparently depends on the employed casting solvent.

**Figure 1:**
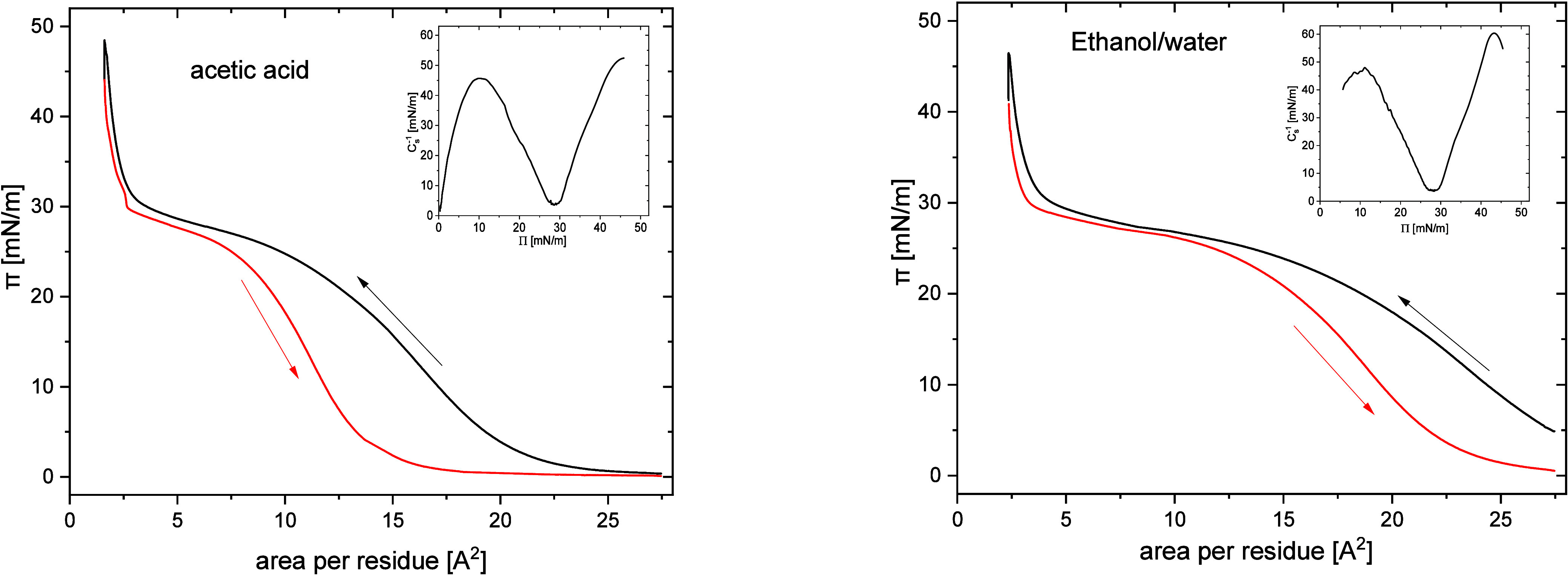
Surface compression–decompression isotherms for the zein monolayers spread from acetic acid (left) and an aqueous ethanol 9:1 mixture (right).

The compressibility modulus $C_{s}^{-1}$ calculated using eq 1 (see insets in Figure 1) confirmed that the phase transition observed around π = 25–30 mN/m is similar for both depositing solvents. The $C_{s}^{-1}$ values in the range 25–50 mN/m both before and after the transition are characteristic of the liquid expanded (LE) phase of a monolayer,^22^ suggesting that the phase transition takes place between two liquid expanded phases (LE-LE).

To shed more light on the unusual LE-LE transition suggested from the surface compression isotherm and compressibility modulus data, the monolayers deposited from acetic acid and ethanol/water were further characterized using neutron reflectometry (NR).

### NR Measurements of Zein Monolayers at the Air/Water Interface

The NR profiles of both zein monolayers were recorded at π = 20, 25, and 30 mN/m during compression and at π = 25 mN/m during the subsequent monolayer expansion (see Figures S1 and S2 in the Supporting Information). The zein monolayers spread at the air/water interface were modeled using two sublayers: the top layer (1) representing the region of the zein monolayer expelled from the water phase, devoid of water, and the bottom layer (2) immersed in water, devoid of air. The fitting parameters were then the thickness of layer 1 (*d*_1_), the volume fraction of air in layer 1 (Φ_1a_), the thickness of layer 2 (*d*_2_), and the volume fraction of water in layer 2 (Φ_2w_). A roughness of 3.5 Å, consistent with the contribution of capillary waves, was applied to all layers.^23^ The zein SLD varied between contrasts according to bulk composition but was not allowed to float in the fitting procedure. The best-fit parameters are collected in Table 1 (see Table S1 for the range of the fitted values). All reflectivity profiles were analyzed using RasCAL software. The uncertainty in the fitting parameters was obtained using Bayesian probability routines available with RasCAL. For simplicity, in the text, every fitted parameter will be referred to as the optimum fit; one should refer to the corresponding tables in the Supporting Information for the associated confidence interval. One interesting aspect is that for all samples, Φ_1a_ was always zero, indicating that the zein expelled from the aqueous phase formed a very compact layer with no defects. The proteinaceous material constituting the layer 1 responded to compression by occupying all the available space, in contrast to many other globular proteins, like BSA or lysozyme,^24,25^ which largely maintain their conformation upon adsorption at the air/water interface. Consequently, their adsorbed layers exposed to air are less compact, with Φ_1a_ > 0.

**Table 1:** Fitting Parameter for Zein Monolayers at the Air/Liquid Interface^a^

**spreading solution**	**Π** (mN m**^–1^**)	***d***_**1**_ **(Å)**	***d***_**2**_ **(Å)**	**Φ**_**1a**_	**Φ**_**2w**_	**Γ** (mg m**^–2^**)
ethanol–water 9:1	20	8.9	0.3	0.0	0.98	1.25 ± 0.16
	25	12.2	0.3	0.0	0.98	1.71 ± 0.11
	30	14.9	46.7	0.0	0.55	5.01 ± 0.36
	25 (exp)	12.2	0.3	0.0	0.97	1.71 ± 0.13
acetic acid	20	8.9	1.4	0.0	0.85	1.28 ± 0.18
	25	11.3	3.8	0.0	0.93	1.62 ± 0.22
	30 (1st)	19.4	46.7	0.0	0.62	5.21 ± 0.50
	30 (2nd)	15.3	54.5	0.0	0.50	5.97 ± 0.29
	25 (exp)	11.6	2.5	0.0	0.98	1.63 ± 0.16

a See Table S1 for the complete set of best-fit ranges for each parameter. Note that the second measurement at π = 25 mN/m for each spreading solvent was realized during the expansion cycle (marked with “exp”). The monolayer spread from acetic acid was analyzed twice in D_2_O, and the results of both experiments are reported separately (1st and 2nd) to highlight the poor reproducibility of the NR measurements at this surface pressure.

The most interesting changes in the NR profiles occurred between 25 and 30 mN/m. The corresponding SLD profiles for the aqueous ethanol-spread monolayers at π = 20, 25, and 30 mN/m (all in compression) and π = 25 mN/m (in a subsequent expansion) are collected in Figure 2. The profiles were superimposed with a sketch of the proposed structure to help visualization. The corresponding NR and SLD profiles for the acetic acid-spread monolayers are collected in Figure S2. The zein monolayer spread from the aqueous ethanol showed no sign of hysteresis, and both measurements at π = 25 mN/m (during compression and expansion) overlapped (Figure 2 and Figures S1 and S2). For π < 25 mN/m, the water-side layer (layer 2) had near-zero thickness with near full hydration, indicating that very little zein material, if any, was immersed in the aqueous phase. The vast majority of the monolayer resided on the air side of the interface (layer 1), forming a very compact layer with thickness increasing from ∼8.9 Å for π = 20 mN/m to ∼12.2 Å for π = 25 mN/m (Table 1 and Table S1). Upon further compression to π = 30 mN/m, the thickness of layer 1 marginally increased to ∼14.9 Å, but the main change was the appearance of a large amount of protein on the aqueous side (layer 2). The thickness of this region increased from practically null to ∼46.7 Å (about three times the layer 1), with Φ_2w_ dropping from almost 100% to ∼55.3% (Table 1). This process resulted in a dramatic increase in the adsorbed amount, Γ (from 1.75 ± 0.11 mg/m^2^ at π = 25 mN/m to 5.14 ± 0.36 mg/m^2^ at π = 30 mN/m), pointing to an important conformational change. The latter happens to be completely reversible, as shown by the similarity of both NR/SLD profiles recorded at 25 mN/m (during compression and expansion, see Figure 2 and Figures S1 and S2). The SLD profile change observed between π = 25 and 30 mN/m suggests that the capacity of layer 1 (air side) to accommodate the protein material is limited, and at a certain point, the excess material must be expelled to the aqueous side of the interface (layer 2). It is currently not possible to speculate whether the protein on the aqueous side would rather form hydrated loops, or if the monolayer breaks up, forming rafts that pile on top of each other, possibly forming nanoscale aggregates immersed in the aqueous phase. The latter picture would agree with the observations of Zhang et al., who showed using AFM that the ethanol-spread monolayer of zein transferred at π = 18 mN/m onto a glass surface was composed of the 30–80 nm nanoparticles.^11^ On the other hand, however, the quasi-reversibility of the phase transition together with quite similar elastic properties of both phases (both assigned as LE according to their compressibility) seems to support rather the formation of a homogeneous structure with highly hydrated protein loops. The maximum thickness of the zein monolayer compressed to π = 30 mN/m (∼6 nm) might correspond to a multilayer of the elongated prisms (13 × 1.2 × 3 nm^3^) in the model of Matsushima et al.,^3^ oriented parallelly to the air/water interface (side-on, see the TOC picture). The rationale behind the proposed side-on orientation is the presence of hydrophilic loops along the longest dimension of the prism (13 nm), unfavorable for any perpendicular (edge-on or end-on) orientation. Analogously, for π < 30 mN/m, the adsorbed layer would consist of a single side-on layer tightly packed on the air side. Such tightly packed prisms would possibly experience some distortion to explain the full packing of such a monolayer (Φ_1a_ = 0). The proposed monolayer–multilayer transition without the prism reorientation would also explain the similar lateral compressibility of both LE phases observed in Figure 1.

**Figure 2:**
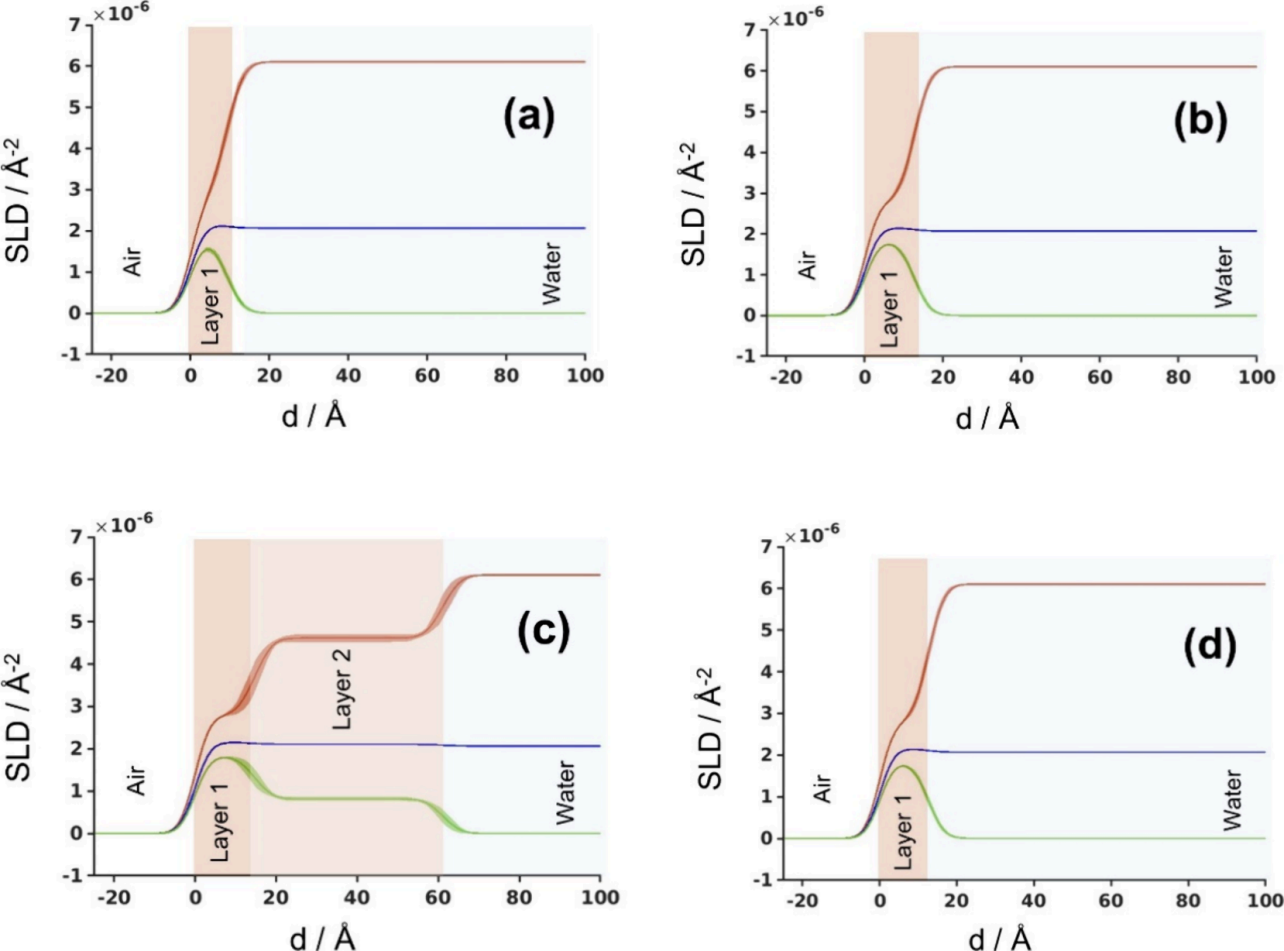
Scattering length density (SLD) profiles for the aqueous ethanol-spread zein monolayers compressed to π = 20 mN/m (a), π = 25 mN/m (b), and π = 30 mN/m (c) and expanded back to π = 25 mN/m (d). Red: D_2_O measurements; blue: zein-matched contrast measurements; green: H_2_O measurements. The SLD profiles are overlaid with a schematic representation of the proposed monolayer structure for the sake of clarity.

Results for the zein monolayer spread from acetic acid are largely similar to those for the aqueous ethanol-spread zein, with one major difference: poorer reproducibility of the NR measurements at π = 30 mN/m. Consequently, the results for all three contrasts at this particular surface pressure could not be cofitted, and the two sets of best-fit results for the acetic acid-spread monolayer at π = 30 mN/m in Table 1 were obtained from two independent measurements in D_2_O. The poor reproducibility of the NR measurements might be related to some kinetic limitations of the phase transition within the acetic acid-spread monolayer, rendering the results very sensitive to the experimental conditions. This hypothesis is corroborated by the appearance of the kink in the expansion branch of the surface pressure isotherm and the higher hysteresis (see above), suggesting a more brittle structure of the acetic acid-spread zein monolayer. Despite the numerical differences, the resulting picture of the monolayer behavior around π = 30 mN/m is quite similar for both spreading solvents. In both cases, the LE-LE phase transition was to a large extent reversible in the range 25–30 mN/m, as also observed in the surface pressure isotherms. The thickness of the layer region in air (layer 1) increased slightly with respect to that at lower π (still maintaining Φ_1a_ = 0). Much more pronounced changes were observed on the water side (layer 2), where the thick ∼5 nm layer formed, with about half of its volume filled with the proteinaceous material.

### NR Measurements of Zein Monolayers at the Solid/Liquid Interface

To further investigate the effect of a spreading solvent on the structure of zein layers, the latter were deposited onto the Si surface from two solvents: ethanol–water (9:1) and acetic acid. Each system was measured in duplicate as each was subsequently tested against 1% SDS or QBS solutions to compare their resistance to detergent activity of the model (bio)­surfactants. A first attempt was made to fit the data using a single protein slab. While the fringe spacing could be mostly described, the fit was still not of sufficient quality. However, the introduction of a cushion layer between the oxide and the main protein layer (the “adhesion layer”) significantly improved the fit quality. When cofitting the D_2_O and H_2_O data, in some cases, we observed a change in fringe spacing: for this reason, the thickness of the H_2_O contrast was fitted independently, but the hydration was changed proportionally to maintain a fixed amount of protein within the layer. The reflectivity profiles are shown in Figure 3 for the aqueous ethanol (a) and acetic acid (c) spread systems (only one repetition is shown). The corresponding scattering length density profiles are also reported for the aqueous ethanol (b) and acetic acid (d) spread systems, along with a superimposed sketch of the proposed structure to help visualization. The fitting parameters for all four layers studied (including those not shown in Figure 3) are reported in Table 2 and Table S2.

**Figure 3:**
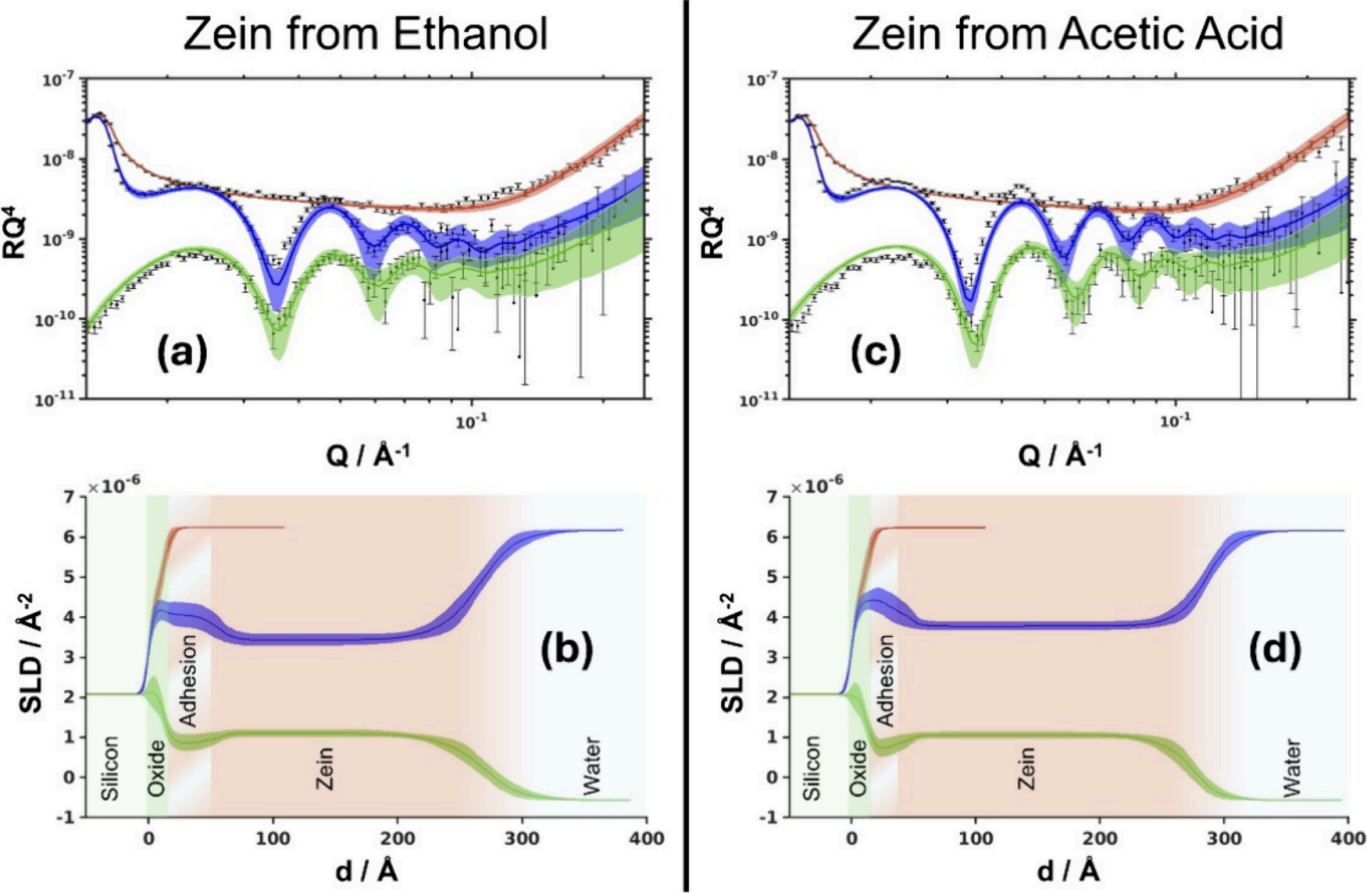
Reflectivity profiles for zein layers spread from (a) aqueous ethanol and (c) acetic acid solutions. The red line represents Si/D_2_O before deposition, whereas the zein monolayer is shown in blue for D_2_O and green for H_2_O. Figures (b) and (d) show the corresponding SLD profiles, along with a superimposed sketch of the proposed interfacial structure.

**Table 2:** Summary of the Fitting Parameters for All Four Zein Layers Studied^a^

spreading solvent	**aqueous ethanol**		**acetic acid**	
Adhesion layer parameters				
adhesion layer thickness (Å)	41.0	37.5	29.0	17.0
adhesion layer hydration (%)	57.5	62.5	61.5	63.0
Main zein layer parameters				
roughness zein-bulk (Å)	27.5	39.3	20.5	23.5
zein layer thickness (Å)	208.5	226.5	241.0	262.5
zein layer thickness (H_2_O) (Å)	207.0	211.5	229.0	225.0
zein layer hydration (%)	27.3	36.5	32.8	44.3
total adsorbed amount (mg m^–2^)	20.16	20.96	24.21	21.37

a See Table S2 for the complete set of best-fit ranges for each parameter. The total adsorbed amount is also shown at the bottom of the table, and a zein density of 1.40 mg/mL was assumed for the calculation.

The first noticeable difference between the zein layers spread from the two solvents is the thickness of the main layer: zein spread from acetic acid is on average about 15% thicker. Although the results are within error bars, the measurements seem to point to a higher amount of adsorbed zein when zein is spread from the acetic acid solution. The adsorbed amount and thickness are significantly higher than those for the corresponding monolayers at the air/water interface, confirming that the spin-coating produced multilayers of zein at the silica surface. The thin adhesion layer at the boundary between silicon oxide and the main zein layer accounts for between 6 and 16% of the overall layer thickness. This layer is more hydrated compared to the main protein layer and may point to a low tendency of zein to adhere to silicon oxide. Although the values are within error, the distribution of thickness suggests that the adhesion layer is somewhat thicker for the system spread from the aqueous ethanol, while the hydration remains virtually identical.

Considering all these points, the picture we can deduce from the measurements shows that the zein layer cast from acetic acid is slightly thicker and shows a stronger adhesion to silicon oxide compared with the system cast from the aqueous ethanol. In contrast to silicon oxide, zein protein is generally hydrophobic, so the partly hydrated adhesion layer probably provides an anchor point for the protein. It should be stressed, however, that all attempts to fit the NR profiles with the adhesion layer composed of a pure aqueous phase (without any proteinaceous material) were unsuccessful, confirming that the adhesion layer was indeed composed of hydrated zein fragments. The latter would probably consist of hydrophilic, e.g., glutamine-rich fragments (loops in Matsushima’s model). Despite being strongly hydrated, the adhesion layer remains sufficiently strong to maintain the protein layer attached to silicon during the aqueous phase exchange (for changing NR contrasts between D_2_O and H_2_O). As will be shown below, it is, however, not strong enough to hold the zein layer in the presence of a strong detergent.

In the next step, the four zein layers, two from acetic acid and two from the aqueous ethanol, were tested against 1% sodium dodecyl sulfate (SDS) and Quillaja bark saponin (QBS) solutions in D_2_O. The two surfactants were chosen as representative synthetic and natural ones, respectively. Not surprisingly, in both zein systems, SDS addition led to the complete removal of zein (Figure S3), in agreement with its known detergent effect on protein layers. The effect of QBS, on the other hand, was highly dependent on the system investigated. While the aqueous ethanol-cast layer was fully removed, the one cast from acetic acid was retained, with NR showing only small changes (Figure 4a). To account for these changes, an additional layer located at the interface between the main zein layer and the bulk aqueous phase containing QBS was introduced to the model (SLD of QBS in D_2_O was taken as 2.80 × 10^–6^ A^–2^), see Figure 4b. It should be stressed, however, that even if the introduction of the layer improved the fit quality, the sensitivity to this layer was hindered by the thick zein layer. The thickness of the adsorbed QBS layer in the range 9.1–32.1 Å (best-fit 15.0 Å) and hydration in the range 39.6–86.6% (best-fit 60.5%) are close to those previously reported for QBS at the air/water interface (19 Å and 65%, respectively).^26^ The results suggest that saponins from QBS would adsorb onto the zein layer cast from an acetic acid in a similar way as at the air/water interface, confirming the zein’s highly hydrophobic nature. The present data do not allow us to verify if the same process preceded the detergent action of QBS on the acetic acid-cast zein layer or that of SDS on both zein layers. The slightly higher resistance of the monolayer spread from the acidic solvent is in line with the previously reported higher values of Young’s modulus of zein films deposited from the aqueous acetic acid dispersions as compared to that from the aqueous ethanol.^1^

**Figure 4:**
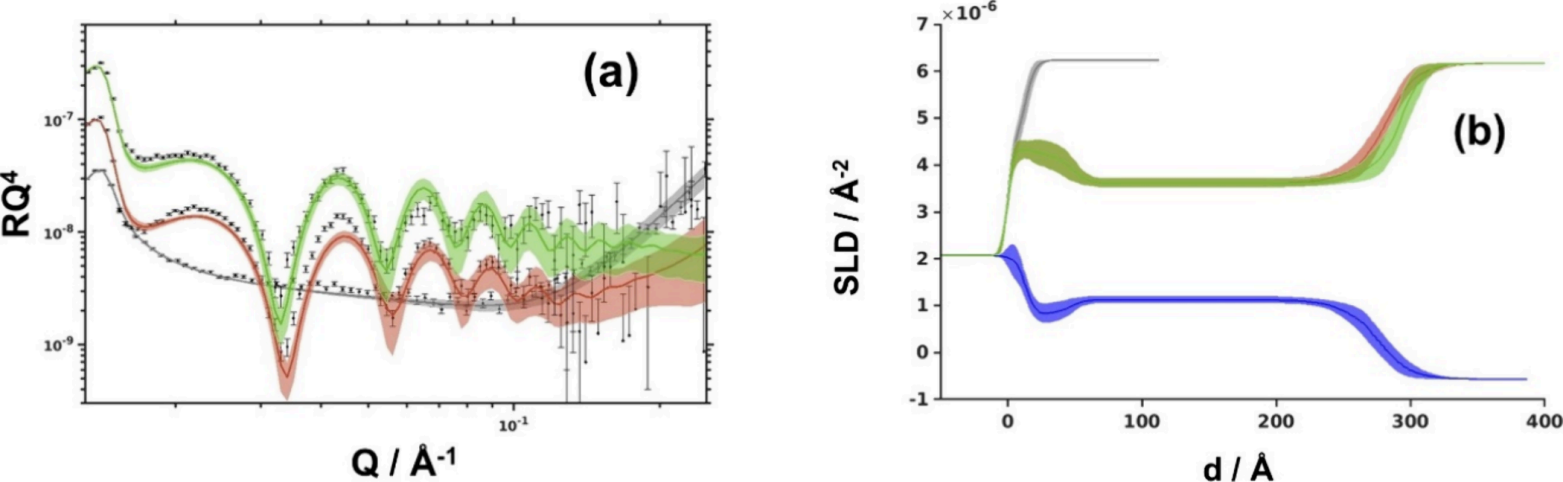
(a) Reflectivity profiles for (bottom to top) silicon oxide (gray), acetic acid-spread zein layer (red), and zein layer after treatment with QBS (green). All measurements were taken against D_2_O as a subphase and are shifted for clarity; (b) SLD profiles for acetic acid-cast zein in D_2_O (red) and H_2_O (blue). The SLD after QBS adsorption is shown in green: the difference is entirely due to the interfacial QBS layer.

### Effect of (Bio)­surfactants on Zein and Its Monolayers

Despite different structures, zein bears some resemblance to keratin, mainly in its poor aqueous solubility, slightly increasing in the presence of anionic surfactants. The increased solubility of zein in aqueous solutions of SDS is also corroborated by the NR tests described above. The anionic surfactant-induced solubilization of zein was found to correlate well with their skin-irritating potential and is now routinely employed for *in vitro* testing of the surfactants and cosmetic formulations.^7^ In order to mimic the effect of the surfactants and saponin-rich plant extracts on keratin present in skin and hair, we employed the zein monolayers spread from both solvent mixtures on Milli-Q water as a subphase. The monolayers were compressed to π_0_ = 30 mN/m,^27^ after which the barriers were stopped, and the subphase was exchanged to simulate the exposure of the protein layer to 1% solutions of the (bio)­surfactants: anionic SDS, cationic CTAB, nonionic Triton X-100, saponins from QBS, and two saponin-rich plant extracts: soapwort and cowherb. As a reference, the intrinsic surface activity of the (bio)­surfactants employed was tested using their respective Gibbs layers (the subphase was exchanged in the absence of a zein monolayer, Figure 5a). During the first minutes of subphase exchange against any (bio)­surfactant, surface pressure of the precompressed zein monolayer slightly decayed for both spreading solvents (Figure 5b,c). In a reference experiment, where the aqueous subphase was exchanged for pure water, the downward trend continued for at least 6000 s (where the experiment was stopped). However, for the saponin-rich plant extracts, a rebound could be observed after about 1 h, resulting in surface pressure at the end of the experiment π_6000_ ≈ π_0_. Much more pronounced changes in surface pressure accompanied the subphase exchange for the synthetic surfactants. The rise of surface pressure started within 15 min from the start of the subphase exchange and was much steeper than in the case of the plant extracts. Given that the hydrodynamic conditions were the same for all samples, the observed differences in the lag times must stem from the adsorption barriers, likely related to the differences in molecular dimensions and hence diffusion coefficients of the low-molecular-weight synthetic surfactants and the medium-molecular-weight saponins. The differences in adsorption kinetics can be also noticed in the reference experiments without the zein monolayer (Gibbs layers of the surfactants and plant extracts, Figure 5a). For water and the plant extracts, even after 6000 s, no steady-state surface pressure values could be achieved, neither for the Langmuir monolayers nor for the Gibbs layers. In contrast, in the case of synthetic surfactants, the surface pressure raised so quickly that in some cases (Triton X-100), the subphase wetted the trough and the barrier walls, causing leakage and prohibiting further monitoring of surface pressure and subsequent surface compression rheology analysis. The latter could only be performed for the monolayers on a subphase exchanged for the biosurfactant solutions (see Table 3 for the surface compression elasticity, *E*, values after 6000 s of subphase exchange (at a 0.1 Hz barrier oscillation frequency) on zein monolayers spread from the aqueous ethanol). As a result of the subphase exchange, *E* values increased with respect to the monolayer on pure water (for all extracts) and decreased with respect to the Gibbs layers (for QBS and cowherb). For the soapwort-penetrated monolayer, surface elasticity was not only higher than that of the same monolayer on water but even higher than that of the soapwort Gibbs layer, clearly pointing to a formation of a mixed interfacial layer of increased mechanical strength.

**Table 3:** Surface Compression Elasticity, *E*, after 6000 s of Subphase Exchange on Zein Monolayers Spread from Aqueous Ethanol^a^

	*E*_6000_ (mN/m)			
subphase	water	QBS (1%)	soapwort (1%)	cowherb (1%)
Gibbs layers		163.9 ± 38.2	32.3 ± 8.0	168 ± 30.1
zein monolayer	25.5 ± 2.4	65.9 ± 19.6	94.1 ± 25.2	69.5 ± 17.0

a Barrier oscillation frequency, 0.1 Hz.

**Figure 5:**
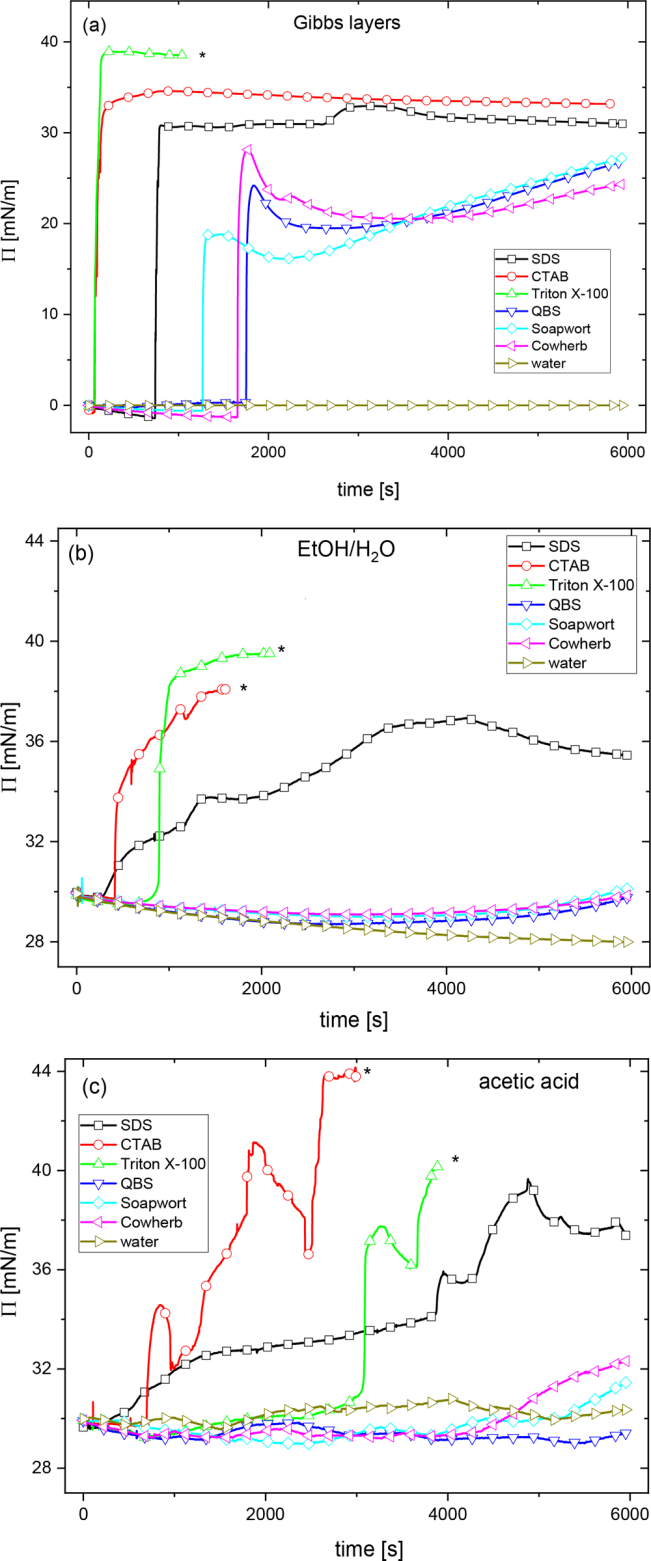
Time course of surface pressure in Gibbs layers (a) and Langmuir monolayers of zein spread from the aqueous ethanol (b) and acetic acid (c) upon introduction of (bio)­surfactants. The asterisk (*) indicates the point where the trough was wetted by the subphase.

Independently of the spreading solvent, the employed zein monolayers were practically unaffected by 6000 s of contact with water or saponin-rich extracts. In contrast, the presence of synthetic surfactants resulted in abrupt changes in surface pressure, suggesting strong interactions with the air–water interface or with the monolayers. For the ones spread from the aqueous ethanol, the π­(*t*) curves for the Triton X-100 and CTAB solutions closely resembled those for the respective Gibbs layers. The short lags observed in the presence of zein monolayers suggest that the latter could only briefly resist the detergent activity of the cationic and nonionic surfactant. Surprisingly, for the anionic SDS, the rise of surface pressure was much less steep, pointing to a possibly different mechanism of zein–protein interactions (Figure 5). The acetic acid-cast monolayers showed much more dynamic behavior upon contact with the synthetic surfactants. The lag times preceding the steep rise of surface pressure were longer than those for the monolayers spread from the aqueous ethanol mixture. They were also longer than those for the corresponding Gibbs layers, which altogether suggests a higher resistance of the acetic acid-spread monolayer to the detergents, also in line with its higher resistance to QBS at the Si/water interface (see NR results above). Moreover, the monolayers spread under acidic conditions remained visually intact for longer times (no trough wetting), even though the surface pressure experienced several jumps for all three tested low-molecular-weight surfactants.

With the exception of Triton X-100, the detergent activity of the (bio)­surfactants investigated using the zein monolayers correlated quite well with their ability to solubilize solid zein in their aqueous solutions. The latter was determined on the basis of an increase in a dry mass of the respective (bio)­surfactant solution after contact with a zein powder (Table 4). Both ionic surfactants (SDS and CTAB) dissolved zein to the highest extent (2.00 and 1.61%, respectively) and caused a significant increase in surface pressure in the monolayer experiments (Figure 5b,c). In both cases, however, the increase was slower than that for the corresponding Gibbs layers (Figure 5a), pointing to some resistance of the zein monolayer and its continuous rather than abrupt displacement by the surfactants. In contrast, a nonionic Triton X-100 showed a negligible ability to solubilize zein (0.10%, comparable to that of the plant extracts, see Table 4), yet clearly being able to displace zein from the air/water surface. Note, however, that in its presence, surface pressure did not rise continuously (like for CTAB or SDS) but rather jumped abruptly, pointing to a one-step detachment instead of continuous displacement. In other words, the detergent activity of Triton against the zein monolayer would rely on its intrinsic surface activity rather than on its interaction with the protein, in contrast to CTAB or SDS. The opposite situation can be observed for the plant extracts because their surface activity being lower than that of the synthetic surfactants is not sufficient to displace zein from the monolayer. Nevertheless, their affinity to zein clearly affected its surface compression elasticity, *E*, in all cases exceeding that observed for zein on pure water (see Table 3). The plant extract components are thus capable of adsorbing onto zein without dissolving it, which is also corroborated by their low ability to solubilize solid zein. A similar adsorption of QBS onto the zein layer cast from acetic acid was observed at the Si/water interface.

**Table 4:** Solubility of Zein in 1% Aqueous Solutions of (Bio)­surfactants

surfactant/extract (1% solution)	zein solubility [%]
SDS	2.00 ± 0.001
CTAB	1.61 ± 0.001
Triton X-100	0.10 ± 0.0004
QBS	0.11 ± 0.001
soapwort	0.04 ± 0.0003
cowherb	0.15 ± 0.0002

## Conclusions

Despite the great interest in zein as a model of keratin in skin-mimicking systems, so far, only a few studies have discussed its behavior in monolayers. In this contribution, we proved that the zein monolayers can be spread from nonconventional solvents, like aqueous ethanol or acetic acid, and compressed up to ∼47 mN/m. The monolayer undergoes a liquid expanded–liquid expanded (LE-LE) phase transition at π ≈ 30 mN/m, confirmed by the surface pressure isotherm and neutron reflectivity (NR). The LE-LE phase transition is well-reversible and involves an expulsion of the well-packed monolayer initially located on the air side of the interface toward the aqueous side. This is accompanied by a significant increase in both the layer thickness (from ∼1 to ∼6 nm) and the adsorbed amount (from ∼1.7 to ∼5.0 mg/m^2^). Using Matsushima et al.’s model of elongated prisms decorated with hydrophilic loops, we propose that the zein monolayer could be formed by the side-on oriented protein Z19/Z22 units (see the TOC picture). At low compression (for π < 30 mN/m), the monolayer would consist of deformed prisms enabling the highly packed structure. Then, the observed unusual LE-LE phase transition might be caused by a reversible monolayer–multilayer transition. The surface pressure response of the monolayers precompressed to π_0_ = 30 mN/m and exposed to 1% solutions of saponin-rich plant extracts (Quillaja bark saponin, QBS, soapwort, and cowherb) was similar to that on pure water as a subphase. However, the surface compression elasticity of the exposed layers clearly proved at least partial penetration of the protein layers by the extract components, resulting in their increased surface elasticity. In contrast, all synthetic surfactants at the same concentration (1%) removed the protein from the monolayer, either by a continuous exchange (anionic sodium dodecyl sulfate, SDS, and cationic cetyltrimmonium bromide, CTAB) or by a one-step orogenic-like detachment (Triton X-100). The bulk zein solubility in 1% aqueous solutions of the biosurfactants confirmed that the latter did not solubilize zein (in contrast to SDS and CTAB) nor displaced it (like in the case of Triton X-100). The zein layers cast on the silicon surface from both solvents were thicker than those spontaneously adsorbed at the air/water interface (∼20–25 nm) and adhered to the Si/SiO_2_ interface via a highly hydrated adhesion layer (∼2–4 nm), in agreement with the generally hydrophobic nature of the protein. As a consequence of this weak adhesion, the silicon-cast zein layers could be easily removed by the model anionic surfactant (SDS) or even by QBS (in the case of the aqueous ethanol-cast layer). Only the acetic acid-cast zein layer resisted the detergent activity of QBS, confirming its higher mechanical strength.

## Supplementary Material



## Notes

Zein’s amino acid sequence from the UniProt database, neutron reflectivity and SLD profiles for zein monolayers spread from ethanol:water mixtures (Figure S1) and acetic acid (Figure S2), neutron reflectivity profiles for silicon/D_2_O after contact with (bio)­surfactants, and ranges of best-fit parameters for Tables 1 and 2 (Tables S1 and S2) (PDF)
