# Supplementary material for: Zein Monolayers: Characterization and Interaction with (Bio)surfactants
Source: Langmuir. 2025 Jul 23;41(30):20174–83. doi: 10.1021/acs.langmuir.5c02426 (PMC12333351; doi:10.1021/acs.langmuir.5c02426)
Supplement: Supplementary file 1 [file la5c02426_si_001.pdf]

Zein monolayers – characterization and interaction with (bio)surfactants

Kamil Wojciechowski[1,2] *, Mario Campana[3], Agnieszka Samel[1], Emilia Baran[1]

1) *Faculty of Chemistry, Warsaw University of Technology, Noakowskiego 3, 00-664 Warsaw, Poland*
2) *Department of Chemistry, University of Warmia and Mazury in Olsztyn, Pl. Łódzki 4, 10-721 Olsztyn, Poland*
3) *ISIS Pulsed Neutron and Muon Facility, Rutherford Appleton Laboratory, Didcot, Oxfordshire OX11 0QX, UK*

*corresponding author kamil.wojciechowski@pw.edu.pl

Supporting Information

Zein's amino acid sequence from UniProt Database (https://www.uniprot.org/uniprotkb/Q41884/entry#sequences):

MAAKIFSILMLLALSACVLDATIFPQYSQAPIAALLPPYLPSMTASVCENPTLQPYRLQQAIAT
SNLPLSPLLFQQSPALSLVQSLVQTIRAQQLQQLVLPLINQVALANLSPYSQQQQFLPFNQL
STLNLAAYLQQQLLPFSQLATAYSQQQQFLPFNQLAALNPAAYLQQQILLPFGQLATTNRAS
FLTQQQLLPFYQQFSANPATLLQLQQLLPFVQLALTNPAAFYQQHIIGGAIF

<table>
<tr><th colspan="3">Zein from Ethanol</th></tr>
<tr><th>Π / mN m⁻¹</th><th>Reflectivity profiles</th><th>SLD profiles</th></tr>
<tr><td>20</td><td>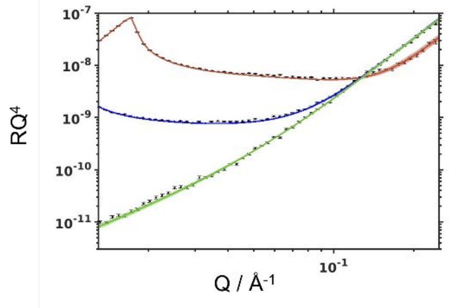
</td><td>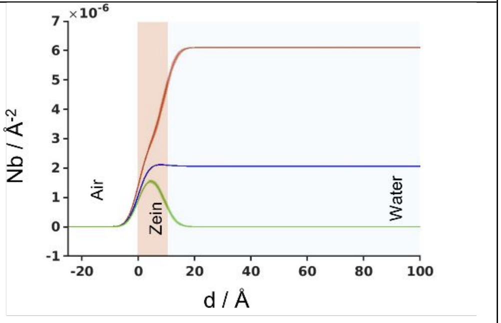
</td></tr>
<tr><td>25</td><td>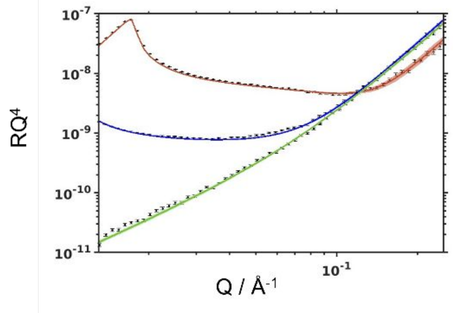
</td><td>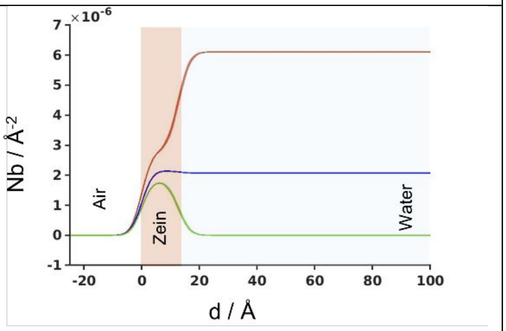
</td></tr>
<tr><td>30</td><td>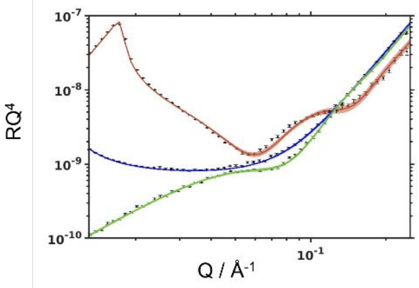
</td><td>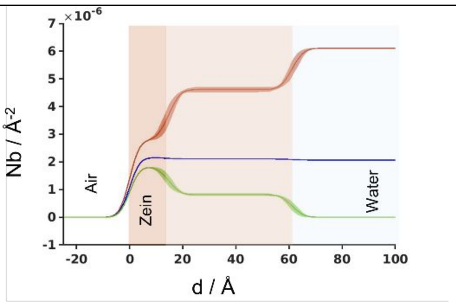
</td></tr>
<tr><td>25
(Expanded)</td><td>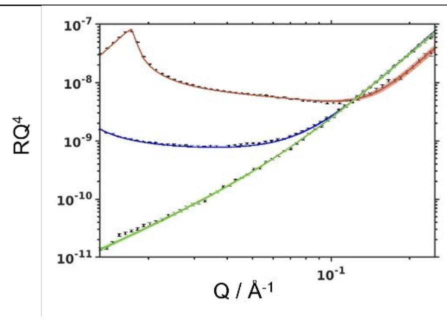
</td><td>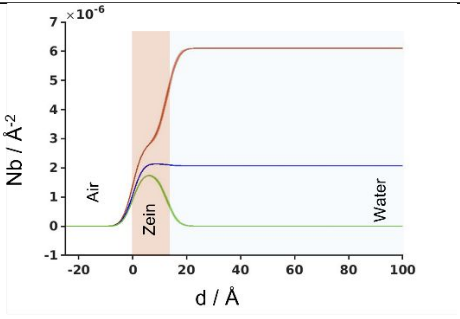
</td></tr>
</table>

Figure S1: Reflectivity profiles (left) and corresponding SLD profiles (right) for zein monolayers at the air/water interface spread from ethanol:water mixtures at different pressures in the isotherm. Red: D_2O measurements, Blue: ZMW measurements, Green: H_2O measurements. The SLD profiles are overlaid with a schematic representation of the proposed monolayer structure for clarity.

Zein from Acetic acid		
Π / mN m^{-1}	Reflectivity profiles	SLD profiles
20	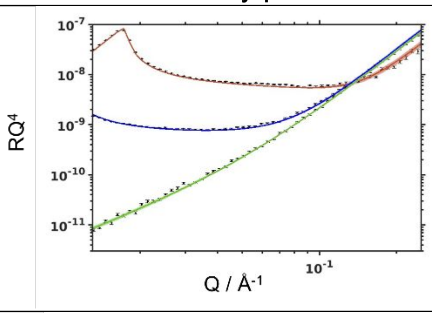	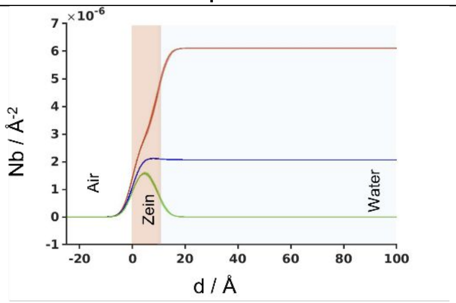
25	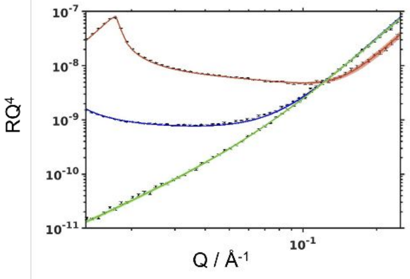	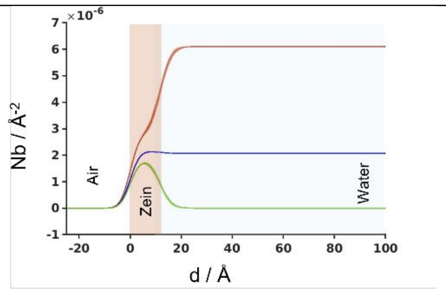
30 (1st)	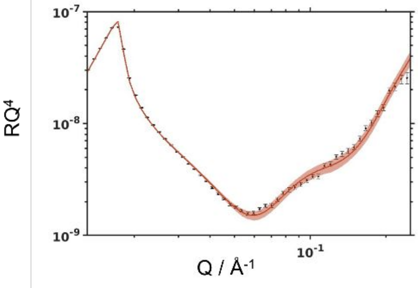	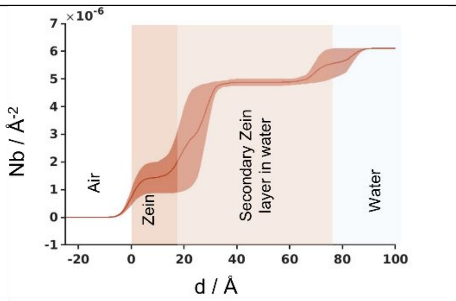
30 (2nd)	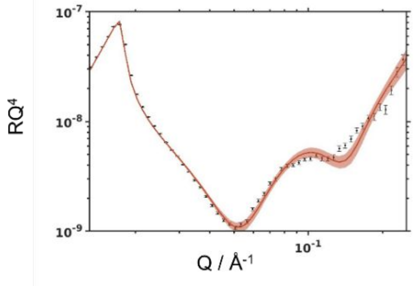	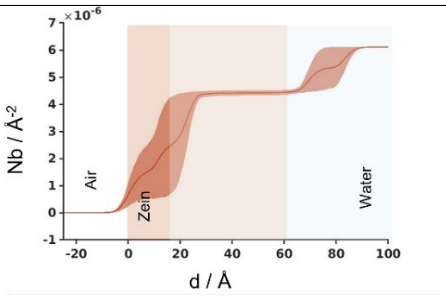
25 (Expanded)	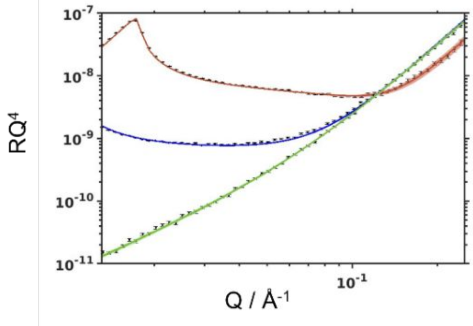	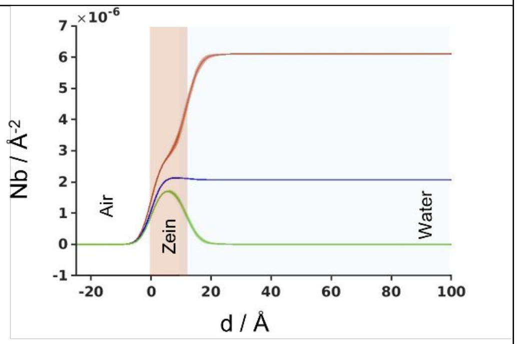

Figure S2. Reflectivity profiles (left) and corresponding SLD profiles (right) for zein monolayers at the air/water interface spread from acetic acid at different pressures in the isotherm. Red: D_2O measurements, Blue: ZMW measurements, Green: H_2O measurements. Because of the lack of reproducibility at 30 mN m^{-1}, the measurement was run in duplicates, both results are shown in table (30(1st) and 30(2nd), respectively). The SLD profiles are overlaid with a schematic representation of the proposed monolayer structure for clarity.

Table S1. Ranges of best-fit parameter (65% confidence interval) for zein monolayers at the air/liquid interface presented in Table 1, together with the number amino acids per nm^2 (AA).

**Spreading solution**	***Π*/ mN m^{-1}**	**d_1 Range / Å**	**d_2 Range / Å**	**Φ_{2w} Range**	**AA /nm^{-2}**
Ethanol / Water 9:1	20	(7.8, 9.1)	(0.0, 2.22)	(0.892, 1.000)	6.7 ±0.9
	25	(11.4. 12.6)	(0.0, 2.4)	(0.920, 1.000)	9.1 ± 0.6
	30	(12.9, 16.1)	(45.4, 46.7)	(0.523, 0.575)	26.7 ± 1.9
	25 (exp)	(11.3, 12.6)	(0.0, 3.5)	(0.897, 1.000)	9.1 ±0.7
Acetic Acid	20	(7.8, 9.3)	(0.0, 3.3)	(0.794, 1.000)	6.8 ± 1.0
	25	(10.0, 11.6)	(0.0, 5.9)	(0.873, 1.000)	8.6 ± 1.2
	30 (1st)	(16.5, 22.0)	(45.0, 48.4)	(0.595, 0.640)	27.8 ± 2.7
	30 (2nd)	(13.6, 17.1)	(53.8, 56.1)	(0.476, 0.518)	31.8 ± 1.5
	25 (exp)	(10.5, 11.9)	(0.0, 4.7)	(0.956, 1.000)	8.7 ± 0.9

Table S2. Ranges of best-fit parameter (65% confidence interval) for zein monolayers at the air/liquid interface presented in Table. 2.

	**Zein spread from Ethanol**		**Zein spread from Acetic acid**	
	**Adhesion layer parameters**		**Adhesion layer parameters**	
Adhesion Layer thickness / Å	(28.3 - 54.2)	(17.9 - 52.0)	(19.9 - 40.2)	(7.2 - 32.6)
Adhesion Layer hydration / %	(46.1 - 60.5)	(58.8 - 70.2)	(52.1 - 67.7)	(58.9 - 78.5)
	**Main zein layer parameters**		**Main zein layer parameters**	
Roughness Zein - Bulk / Å	(16 - 33.8)	(27.7 - 42.0)	(11.7 - 24.9)	(18.8 - 29.0)
Zein Layer thickness / Å	(199.0 - 227.0)	(211.0 - 251.0)	(229.0 - 252.0)	(253.0 - 286.0)
Zein Layer thickness (H_2O) / Å	(199.0 - 227.0)	(199.0 - 217.0)	(220.0 - 241.0)	(207.0 - 235.0)
Zein Layer hydration / %	(24.3 - 30.8)	(34.9 - 43.6)	(28.5 - 34.7)	(42.1 - 47.1)
Total adsorbed amount / mg m^{-2}	(18.59, 22.52)	(18.96, 24.48)	(22.78, 26.05)	(20.12, 24.01)

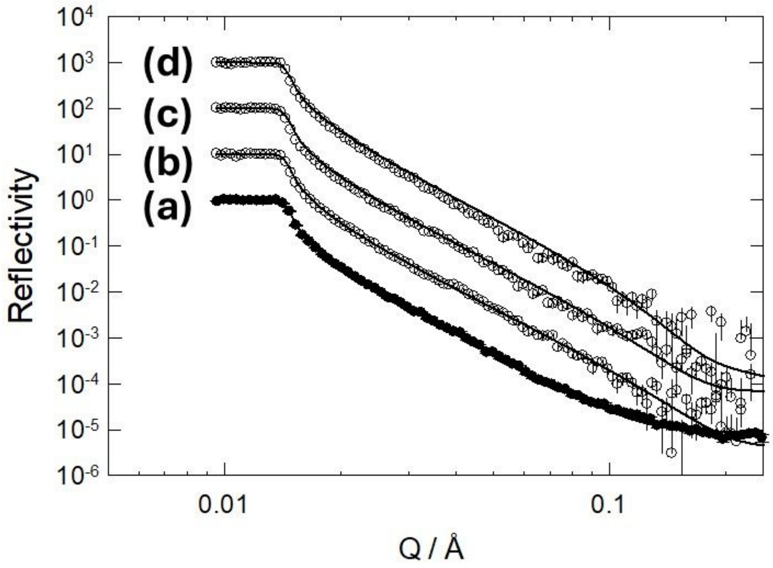

Figure S3. Reflectivity profiles for Silicon/D_2O (a) and for systems where surfactant-induced zein removal was observed: ethanol-cast zein with SDS (b), acetic acid-cast zein with SDS (c), ethanol-cast zein with QBS (d).
